# An Innovative Electronic Health Toolkit (Our Whole Lives for Chronic Pain) to Reduce Chronic Pain in Patients With Health Disparities: Open Clinical Trial

**DOI:** 10.2196/14768

**Published:** 2020-03-30

**Authors:** Paula Gardiner, Salvatore D'Amico, Man Luo, Niina Haas

**Affiliations:** 1 Department of Family Medicine and Community Health University of Massachusetts Medical School Worcester, MA United States; 2 Department of Family Medicine Boston Medical Center Boston, MA United States; 3 BrightOutcome Buffalo Grove, IL United States

**Keywords:** minority health, internet, Web-based, complementary therapies, mindfulness-based stress reduction

## Abstract

**Background:**

Chronic pain affects millions of Americans. Our Whole Lives, an electronic health (eHealth) toolkit for Chronic Pain (Our Whole Lives for Chronic Pain [OWLCP]), is a mind-body chronic pain management platform that teaches self-management strategies to reduce pain impact and pain medication use.

**Objective:**

The primary goal of this study was to evaluate the feasibility of OWLCP in reducing pain impact and pain-related outcomes.

**Methods:**

We conducted a pre-post clinical study (2 cohorts) to assess the feasibility of OWLCP usage among low-income patients with chronic pain. Outcome data, collected at baseline and 9 weeks, included Patient-Reported Outcomes Measurement Information System (PROMIS-29), pain self-efficacy, and pain medication use. In the statistical analysis, we used descriptive statistics, logistic regression, linear regression, and qualitative methods.

**Results:**

Among the enrolled 43 participants, the average age was 50 years, (39/43) 91% were female, (16/43) 37% were black, and (7/43) 16% were Hispanic. From baseline to follow-up, the PROMIS measures showed a reduction in depression (*P*=.02), pain interference (*P*=.003), and average pain impact score (*P*=.007). Pain self-efficacy increased ((*P*<.001), whereas opioid use had a 13% reduction (*P*=.03).

**Conclusions:**

The eHealth chronic pain management platform, OWLCP, is a potential tool to reduce the impact of chronic pain for low-income racially diverse populations.

## Introduction

In the United States, more than half of all adults experience pain in any given year [1]. Chronic pain may be defined as pain that persists past normal tissue healing (>3 months) [2]. It is characterized by substantial suffering and associated with other comorbidities, such as insomnia, depression, fatigue, lowered mobility, and reduced quality of life [2]. Patients with chronic pain mostly receive care during hurried visits to the primary care provider (PCP) where they are prescribed pharmacological treatments (eg, opioids and medications) despite mixed evidence of their efficacy and increased risk of potentially dangerous side effects, including addiction and death [3-6]. Even when these treatments are effective in reducing pain, they may not improve mental and functional status and may actually increase depression [7-9]. There is a need for easy access of evidence-based nonpharmacological treatment options to help patients with chronic pain.

The impact of chronic pain is particularly severe in populations with racial and socioeconomic disparities who receive less patient education, surgery, and specialty referrals [10-14]. Health disparities in chronic pain treatment substantially impact the patients’ ability to work and function [12,15]. The reduced use of nonpharmacological options such as mindfulness-based interventions (MBIs; ie, meditation and yoga) by low-income patients is attributed to limited insurance coverage, therapies not being offered to them as a treatment option, structural barriers such as transportation, or lack of access to these options in their neighborhoods [16-19].

The internet and mobile technology is an accessible, convenient, and time-saving method to deliver health interventions [20,21]. Ziebland et al [22] demonstrated that people living with chronic pain are increasingly using the Web to find information, support, reassurance, encouragement, and practical advice for self-management. They also use the Web to compare experiences of treatment and offer advice and support to others. Chronic pain interventions delivered via technology are increasing because of factors such as acceptability to increase social connection, convenience for patients, and the ability to interact at one’s own pace at home [23-26]. For example, internet delivery can make participation in a Web-based nonpharmacological skill acquisition intervention possible when it otherwise would not have been due to pain flares or reduced mobility [26]. In addition, the flexibility of internet delivery can be appealing for people who are busy managing appointments and treatments, those who want to bypass barriers related to cost and insurance coverage and time commitments for in-person treatment, and those who are reluctant to engage in in-person group interventions [27-29].

As of 2018, US technology trends indicate that 67% of people who earn less than US $30,000, 77% of Hispanic adults, and 75% of African Americans own a mobile phone [30]. Among the clinical literature on technology-delivered interventions, few studies exist on racially diverse and low-income patients with cancer, HIV, or obesity [31-34]. This also applies to racially diverse students and low-income patients with chronic pain [35]. Yet, mobile health is a promising area for health education and intervention delivery in health-disparate communities.

### Development of Our Whole Lives 1.0

The intervention, Our Whole Lives (OWL 1.0), an eHealth Toolkit, was developed during a Patient Centered Outcomes Research Institute (PCORI) Contract AD 1304-6218/ClinicalTrials.gov ID NCT02262377 in 2014 to 2017 [36,37]. This randomized controlled trial (RCT) tested an Integrative Medical Group Visit (IMGV) care model for the usual care in low-income racially diverse patients with chronic pain and depression [36]. The IMGV incorporates key principles and practices of mindfulness adapted for patients who are racially and culturally diverse and have low health literacy levels [38]. The IMGV curriculum introduces patients to the fundamentals of evidence-based integrative medicine such as nutrition, lifestyle, stress reduction, exercise, and massage [39-41]. During the 21-week RCT study, participants had access to OWL as an adjunctive patient education website for the IMGV.

The user interface, visuals, videos, scripts, and resource pages for OWL 1.0 were created with input from a patient advisory group (PAG; patients with chronic pain and depression) and beta testing of IMGV socioeconomically and racially diverse patient cohorts (~20 patients) [42,43]. OWL’s content was designed for patients with low health literacy (grades 5-8) and has been adapted for a diverse patient population by ensuring that the images of patients on the site are representative of the diverse and vulnerable population in the study, for example, visual images and pictures of patients from diverse racial and ethnicity backgrounds on the website. OWL mirrored the curriculum taught in an IMGV including mindfulness exercises and self-management home practices, interactive self-monitoring and self-directed learning, as well as social support through interactions on community blogs. It allowed the participants to access each session one at a time on a weekly basis. The participants were encouraged to participate by commenting on each video, audio, or other experiential activities and track their progress.

**Figure 1:**
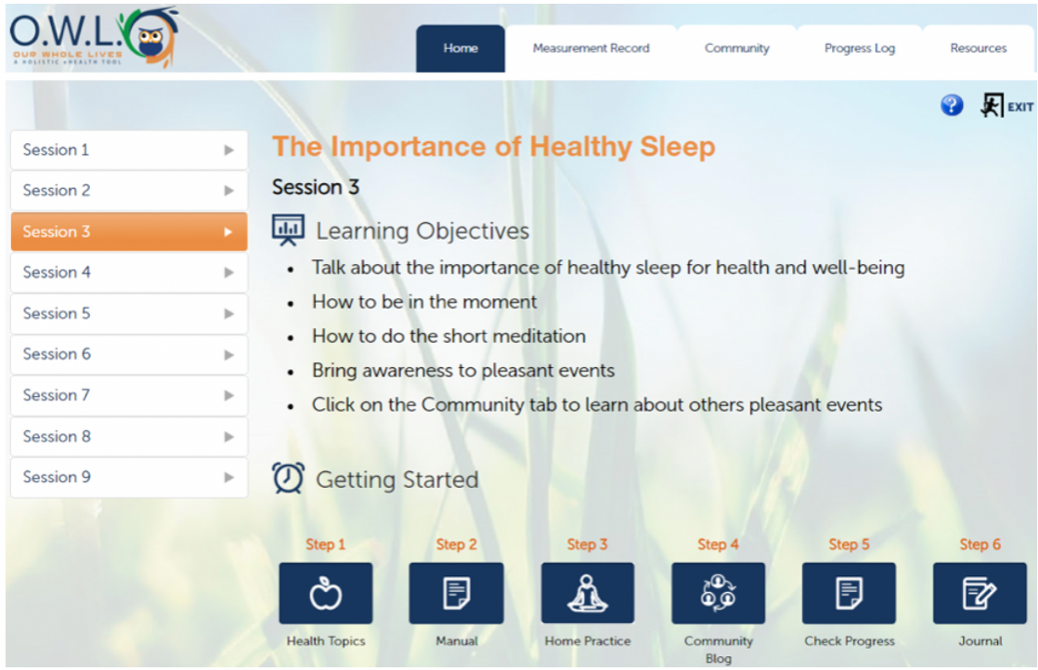
Screenshot of the Our Whole Lives for Chronic Pain (OWLCP) website.

This pre-post clinical trial was conducted to test the second-generation OWL (version 2; Figure 1), which we will refer to as OWL for Chronic Pain (OWLCP) outside of an in-person IMGV to see if the application is feasible to use as a stand-alone intervention. The main outcomes were pain impact (such as pain severity, pain interference, and physical function) and pain-related outcomes (eg, depression, anxiety, fatigue, sleep disturbance, ability to participate in social roles and activities, pain self-efficacy, and pain medication use). Finally, to understand how OWLCP potentially changes behavior, we used the Health Education Impact Questionnaire (HEIQ) to look for changes in health-directed behavior, positive and active engagement in life, social integration and support, and emotional distress [44]. Using these outcomes, we evaluated pre-post effects and estimated effect sizes.

## Methods

### Setting

This study was held at Boston Medical Center (BMC), a private, not-for-profit, academic medical center and the largest safety net hospital in New England. BMC is primarily funded by charities or the government. Approximately 70% of the patients come from underserved populations, such as low-income and older adults, who rely on government payors such as Medicaid, the Health Safety Net, and Medicare for their coverage; 32% do not speak English as a primary language.

### Study Design

This prospective clinical trial enrolled participants with chronic pain between October 2016 and January 2018. We conducted two 9-week cohorts of approximately 40 patients with chronic pain.

### Recruitment and Enrollment

Inclusion criteria were as follows: chronic pain ≥4 on a 0 to 10 pain scale for at least 12 weeks [34,35]; older than 18 years; and the ability to provide informed consent and understand website information in English. Exclusion criteria were as follows: a major medical event or another life event that would interfere with their ability to use the internet and participate in the intervention; not currently having access to the internet; not having an internet-enabled device to access the website; pregnant or planning to become pregnant in the next 3 months; and active substance use of alcohol, cocaine, or heroin.

Recruitment flyers were placed in BMC’s primary care outpatient clinics, local Young Men’s Christian Association (YMCA) and community organizations in the Boston area. Both men and women attend the YMCA, and it acts as a local community resource. Research assistants (RAs) reached out to PCPs, either by attending relevant provider meetings per department (ie, internal medicine and family medicine) or electronically notifying them of this study through an electronic medical record. When a PCP identified potentially eligible participants, they gave the participant a pamphlet about the study; thereafter interested participants contacted the study so they could be considered for the study. Study staff followed up with participants to determine interest and eligibility. Participants also could self-refer themselves to the study to be screened for study enrollment.

If an individual met the specified criteria, they were invited to meet in person at BMC with the RA. They were invited to review and sign the informed consent and collect baseline data. The RA provided clear detailed information about what the study involved. If the participant was unable to come to BMC in person, the consent and initial visit process was conducted over the phone with the RA. Verbal consent was given over the phone; however, informed consent was signed when the participant met with the RA in person.

### Intervention Our Whole Lives 2.0

On the basis of the feedback from the RCT participants and the PAG, OWL, an electronic health toolkit, for Chronic Pain 2.0 (OWLCP) was developed in 2017. OWLCP is the version being tested during this feasibility trial. OWLCP is a password-protected internet-based platform stored on a server compliant to the Health Insurance Portability and Accountability Act; this server could be accessed with a tablet, computer, or mobile phone. This website provided interactive self-monitoring (ie, pain, mood, and medication use), self-directed learning (ie, health topics, mindfulness, movement, and nutrition), and social support (ie, online community forum). Table 1 includes session names, home practice assignments (each ~20 min), themes, and activities. Changes from version 1 to version 2 included the following: revision of curriculum webpages from PDFs to interactive webpages, removing session 10, and adding a pain medication-use tracking tool.

**Table 1:** Our Whole Lives for Chronic Pain website curriculum.

Title of session	Home practice	Theme or activity
Online orientation	N/A^a^	Awareness of breath meditation, ground rules, introduction to mindfulness
Our reactions to stress	BS^b^	Nonpharmacological approaches to stress
Our bodies and healthy sleep	BS, M^c^	Nonpharmacological approaches to sleep
Movement and food as medicine	Alternate BS/CY^d^; M 6 of 7 days	Movement and healthy eating skills
Our bodies’ response to pain	Alternate BS/CY; M 6 of 7 days	Nonpharmacological pain approaches to pain management
Our bodies and inflammation	Alternate BS/CY; M 6 of 7 days	Nonpharmacological approaches to treating inflammation
Our bodies and depression	Alternate BS/CY; loving kindness meditation 6 of 7 days	Nonpharmacological approaches to depression and challenging communications
Understanding the role of food in our body	Choice of BS, CY, M, or loving kindness meditation	Mindful eating
Wellness review	N/A	Wellness review

^a^N/A: not applicable.

^b^BS: body scan.

^c^M: meditation.

^d^CY: chair yoga.

OWLCP’s functions include the following: (1) a daily measurement record (present mood, physical state of the body, and daily medication use); (2) a monitored community blog on which participants post their thoughts and respond to prompts for each session, and the blog is monitored daily by an RA or a clinician; and (3) home practice progress log where the participant may track what mind-body practices they completed (awareness of breath [AOB] meditation, sitting meditation, loving kindness meditation, chair yoga, and body scan). Each mind-body practice was recorded by a certified yoga or meditation teacher and approximately lasts 20 min (audio or video recordings).

OWLCP contains 10 videos that discuss health topics such as prevention and management of pain and associated conditions (such as stress reactivity, insomnia, poor nutrition, inflammation, and depression). Participants are taught to practice principles of mindfulness (AOB meditation, sitting meditation, loving kindness meditation, chair yoga, and body scan) at each session. Patients are encouraged to interact with OWLCP by commenting in an open text box after each video, audio, or other experiential activity to monitor their progress with home practice, such as their mood, pain, and pain medication use, and to choose resources relevant to them. Participants could review all or part of completed modules, earn puzzle pieces and checkmarks by completing audios and videos and selected tasks (eg, practices), and self-monitor (ie, view tables showing progress in mind-body activities, pain, and mood).

OWLCP’s resources library (mind-body resources, low-cost recommendations for nonpharmacological treatments, poetry, community resources, and tips for health eating and recipes) provides a range of chronic pain self-management practices. The RA and primary investigator (PI) monitored the use of the platform, posted questions to facilitate conversations on the community page, and answered any relevant questions. In addition, the participants were given access to a private journal.

For this study, we held two in-person group orientations for participants on how to navigate the OWLCP website. During the orientation, a clinician (PG), assisted by an RA, demonstrated how to use the OWLCP system, log on, navigate through the sessions, complete self-assessments, and interact on the community blog page. Participants had continuous access to OWLCP for 9 weeks. After the orientation, RAs called all participants at weeks 1 and 4 to assess for adverse events, remind them to log on to the website, and check in about technology concerns or problems. Week 4 included a midpoint survey that measured satisfaction and the number of times the participant interacted with different website features (video, audios, webpages, and blog).

### Data Collection

Demographics included the following: age, sex, race, ethnicity, primary language, education level, employment status, and yearly household income. Race was categorized into black/African American, white, or Other. Primary language was dichotomized into English and Non-English. Education level was categorized into high school/Generalized Education Development (GED) or less, some college or associates/no degree, and college graduate/postgraduate. Employment status was categorized into working full-time/part-time, unemployed/retired, and sick leave/disability.

The following information was collected through self-reported questionnaires at baseline and 9 weeks: Patient-Reported Outcomes Measurement Information System (PROMIS-29) [45], HEIQ Version 2.0 [44], pain self-efficacy, Perceived Stress Scale (PSS), pain medication use, and Attitudes Toward Computers Questionnaire (ATCQ) [46].

The PROMIS-29, a 29-item measure, assesses 7 domains: anxiety, depression, fatigue, pain interference, physical function and sleep disturbance, the ability to participate in social roles and activities, and pain intensity [47]. Each domain is scored separately with options ranging in value from 1 to 5, except for the pain intensity item, which ranges in value from 0 to 10. The pain impact score ranges from 8 to 50, which is a summed score of physical function, pain interference, and pain intensity. Higher score means that the patient is more impacted by pain. PROMIS has been validated in low-income racially diverse patients [47]. HEIQ Version 2.0, an instrument for the comprehensive evaluation of patient education programs, is a 40-item survey separated into 8 domains: positive and active engagement in life, health-directed behavior, skill and technique acquisition, constructive attitudes and approaches, self-monitoring and insight, health services navigation, social integration and support, and emotional well-being. HEIQ uses a 5-point Likert-type scale ranging from *strongly disagree* (1) to *strongly agree* (5). Domain scores are calculated by adding the score of items within scales and dividing the sum by the number of items in a particular scale; therefore, all domain scores range between 1 and 4. Higher scores indicate higher levels of self-management ability, with the exception of emotional distress where higher scores indicate more distress [44].

Pain self-efficacy was measured with the Pain Self-Efficacy Scale. It is a sum of 10 items, each rated on a scale of 0 to 6. Higher scores indicate higher levels of confidence in self-managing pain [48]. PSS measures the degree to which situations in one’s life are perceived as stressful in the past month. PSS is a sum of 4 items each with a 0 to 4 scale. Items 2 and 3 are reversely scored. Higher scores indicate higher levels of perceived stress [48].

Data for self-reported pain medication use in the past 7 days were recorded at baseline and 9 weeks. Medications were categorized as either opioids, nonsteroidal anti-inflammatory drugs (NSAIDs), or other medications. Opioid includes the following: MS-contin, vicodin, oxycodone, oxycontin, percocet, tramadol, tylenol with codeine #3, and other medications (suboxone, codeine, and methadone). NSAIDs include ibuprofen, naproxen, aspirin, and other (nabumetone, ketoprofen, celecoxib). Miscellaneous/other medication includes acetaminophen, cyclobenzaprine, gabapentin, and other medications (pregabalin, diazepam, Biofreeze, nortriptyline, lidocaine, naratriptan, Cymbalta, magnesium, tizanidine, baclofen, and valium).

ATCQ assesses seven dimensions of attitudes toward computers. We used two items of the seven dimensions: comfort and efficacy. All items are in a 5-point Likert scale format, with response options ranging from strongly disagree to strongly agree [46]. The OWLCP platform tracks the number of log-ins and minutes for all activities such as watching videos, body scans, chair yoga, and meditations. All blog entries were collected and categorized.

Surveys were administered either in person or on the phone or via an email invitation through REDcap (v9.1.0 Vanderbilt University)—a password-protected research tool. Participants received US $50.00 for their involvement with the cohort study. The initial US $25.00 was disbursed after completion of the 9-week survey, and the other US $25.00 was given after the participants attended a focus group. These funds were disbursed using BMC/s Clincard system.

### Data Analysis

Descriptive statistics were used to analyze survey information and adverse events. Means and SD as well as frequencies and percentages were calculated for demographic characteristics. Means and SDs were also calculated for PROMIS-29, HEIQ, PSS, Pain Self Efficacy Scale, medication use, and ATCQ at baseline and 9 weeks. In terms of OWL usage data, we tracked and summed the average and total number of minutes of mind-body practice, the number of times participants blogged, the number of times participants used the journal, and the time spent on OWLCP website. We also summarized quotes from the blog.

For the PROMIS-29 questionnaire, the scores for 7 subscales, except for *pain intensity* subscale, were converted into standardized *t* scores and SDs and compared with a national distribution of standardized *t* scores and SDs (mean 50, SD 10). The 95% confidence intervals (CIs) were calculated for each subscale in PROMIS-29. Higher scores are associated with better outcomes for physical function and satisfaction of social role. For physical function, the scores ranged from 1 (least difficult) to 5 (most difficult). Therefore, we reverse-coded for questions for physical function to make it a positive subscale. The questions for satisfaction of social role were positively scored. Lower scores are associated with better outcomes for anxiety, depression, fatigue, sleep disturbance, and pain interference.

Finally, pain intensity was scored using a 0 to 10 scale. Means and SDs were calculated for this variable. A lower score was associated with a better outcome. Pain impact score ranged from 8 to 50, which is a summed score of physical function, pain interference, and pain intensity. A higher score meant being more impacted by pain [49].

In HEIQ, the effect sizes and changes in percentages were calculated based on the scoring instructions for HEIQ. Means for each subscale at baseline and follow-up were calculated, and the group-change effect size was calculated from the mean change.

To compare the results between baseline and follow-up, we applied *t* test and multivariate regressions. For continuous outcomes with normal distributions, we used the paired *t* test. For nonparametric continuous outcomes in PROMIS-29 and ATCQ, we applied longitudinal linear regressions with Poisson model and a time predictor to calculate the *P* values. For binary outcomes in medication use questionnaire, the longitudinal logistic regression was used to compare baseline and follow-up by calculating the odds ratios (ORs) and CIs. We used multiple imputation method for missing data. For the blog posts from the OWLCP platform, the posts were categorized by themes. All quantitative analyses were conducted using SAS 9.3. (SAS Enterprise Miner 13.1, SAS Institute Inc). For the qualitative data collected, all blog posts were individually analyzed and coded by 2 RAs using modified grounded theory. The blog posts were independently coded, and a codebook was generated. The PI primarily served to resolve differences found between the 2 initial coders.

This study was approved by the BMC Institutional Review Board.

## Results

**Figure 2:**
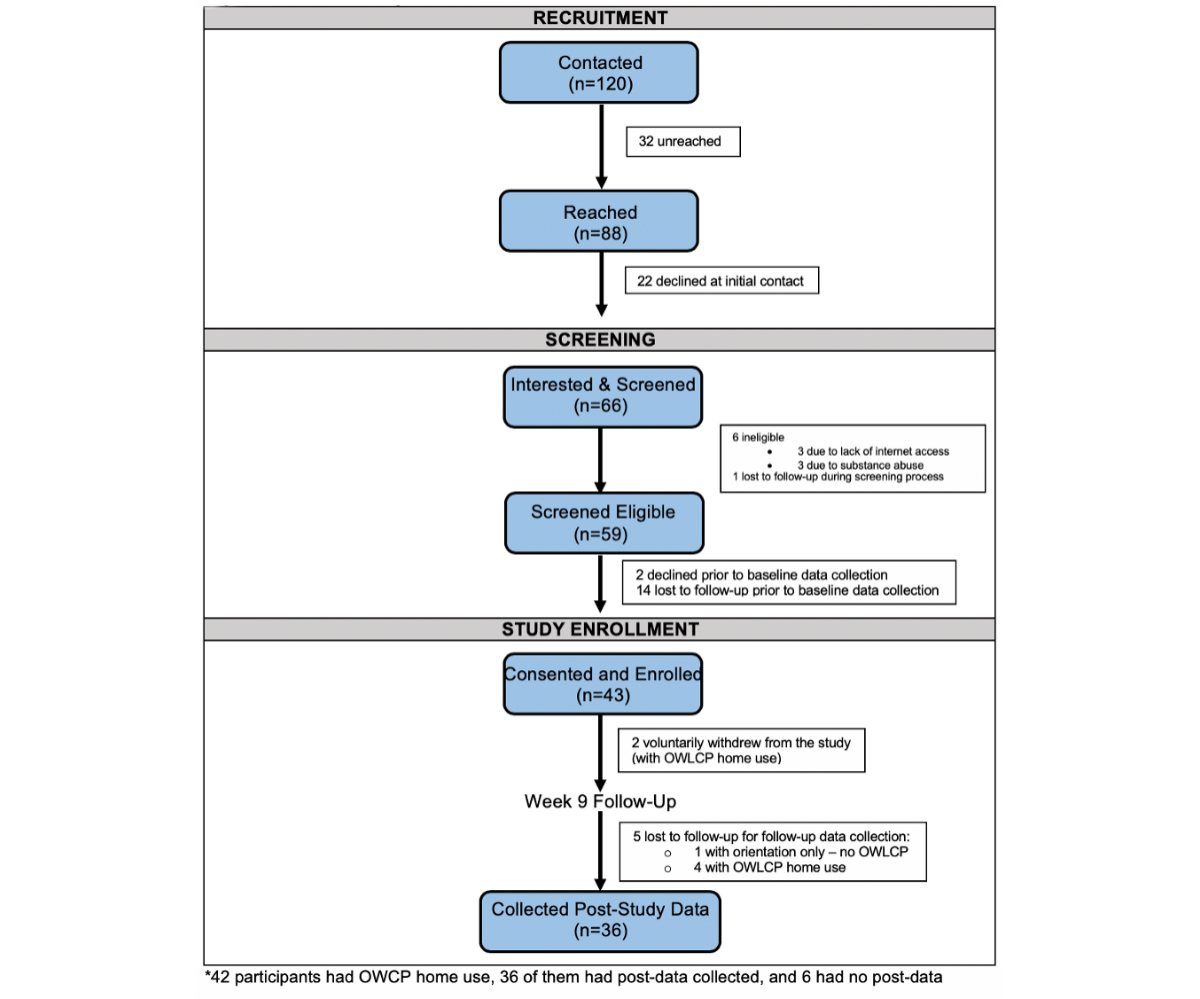
CONSORT diagram.

The study flow, screening, and study enrollment are shown in Figure 2. For possible participation in this study, the study team contacted a total of 120 participants by phone who were either self-referred or referred by a clinician. Of the total, 66 participants agreed to be screened, 59 were eligible, and 43 were enrolled. Of these, 7 participants were screened and found ineligible for the study (Figure 2). After enrollment (n=43), 2 participants voluntarily withdrew from the study, and 5 participants were lost to follow-up. Of these, 36 participants completed follow-up data collection (17% did not complete study). Specifically for the two cohorts, of the 18 participants who started cohort 1, 17 completed, whereas of the 25 participants who started cohort 2, 19 completed.

Table 2 lists all demographics factors. At baseline, the average age was 50 years, most participants were female (39/43, 91%), 37% (16/43) identified as black/African American, and 30% (13/43) identified as white. Of these, 23% of the participants (10/43) completed some high school. More than half of participants had some college degree or higher (33/43, 77%). Most participants were either unemployed/retired or on sick leave/disability (28/43, 65%). Approximately 26% (11/43) of the participants had a yearly household income of US $10,000 or less.

**Table 2:** Demographic characteristics.

Variables		Total (N=43)	Cohort 1 (n=18)	Cohort 2 (n=25)	*P* value
Age (years), mean (SD)		50.4 (12.6)	47.7 (11.9)	52.4 (12.9)	.23
**Sex, n (%)**					
	Female	39 (91)	16 (89)	23 (92)	>.99
**Race, n (%)**					.46
	Black/African American	16 (37)	8 (44)	8 (32)	
	White	13 (30)	6 (33)	7 (28)	
	Other^a^	14 (33)	4 (22)	10 (40)	
**Hispanic/Latino, n (%)**					
	Yes	7 (16)	2 (11)	5 (20)	.68
**Primary language, n (%)**					
	English	40 (93)	17 (94)	23 (92)	>.99
**Education level, n (%)**					.74
	High school/generalized education development or less	10 (23)	5 (27)	5 (20)	
	Some colleges or associates	16 (37)	7 (39)	9 (36)	
	College or associate graduate, postgraduate	17 (40)	6 (33)	11 (44)	
**Employment status, n (%)**					.20
	Working full time or part time	15 (35)	6 (33)	9 (36)	
	Unemployed/retired/other^b^	11 (25)	7 (39)	4 (16)	
	On sick leave/disability	17 (40)	5 (28)	12 (48)	
**Yearly household income, n (%)**					.22
	US $10,000 or less	11 (26)	6 (33)	5 (20)	
	US $10,001-US $90,000	16 (37)	4 (22)	12 (48)	
	Refused/do not know	16 (37)	8 (44)	8 (32)	

^a^Other includes Native American (n=2), refused to answer (n=1), and other races (n=11).

^b^Other includes student (n=3) and other working status (n=3).

Table 3 lists the baseline and 9-week PROMIS-29 average t scores, means, SD, and 95% CI. At baseline, participants’ physical function was 12 points lower than the national average standard t score (t score mean 38.2, SD 2.30). Table 3 shows a comparison of PROMIS-29 between baseline and follow-up. Depression decreased from baseline (t score mean 55.8, SD 2.87) to follow-up (t score mean 52.4, SD 3.40). This change was statistically significant (*P*=.02) with a large effect size (*d*=1.08). Satisfaction of social role increased from baseline (t score mean 40.1, SD 2.51) to follow-up (t score mean 42.9, SD 2.6; *P*=.09, d=1.10). Pain interference also showed a significant decrease (*P*=.003) with a large effect size (*d*=1.67) from baseline (t score mean 66.7, SD 2.20) to follow-up (t score mean 63.1, SD=2.10). Pain intensity decreased from baseline (mean 7.0, SD 1.48) to follow-up (mean 6.5, SD 2.22; *P*=.07, d=0.27). The decrease of pain impact was also statistically significant (*P*=.007) with a medium effect size (*d*=0.42) from baseline (mean 33.95, SD 7.4) to follow-up (mean 30.61, SD 8.53). There was no significant change from baseline to follow-up for physical function, anxiety, fatigue, and sleep disturbance. 

**Table 3:** Patient-Reported Outcomes Measurement Information System-29 results: baseline and 9 weeks.

Item names		Baseline total (N=43)		9-week total (N=36)		*P* value	Effect size
		*t* score (*df*=42), mean (SD)	95% CI	*t* score (*df*=35), mean (SD)	95% CI		
**Subscales (0-100)**							
	Physical function	38.2 (2.3)	33.6-42.7	38.0 (2.2)	33.7-42.4	.80	0.09
	Anxiety	57.0 (3.4)	50.4-63.6	56.3 (3.4)	49.9-63.2	.98	0.21
	Depression^a^	55.8 (2.9)	50.1-61.4	52.4 (3.4)	46.1-59.3	.02^b^	1.08^c^
	Fatigue	59.9 (2.6)	54.9-64.9	57.7 (2.5)	53.0-62.9	.25	0.87^c^
	Sleep disturbance	60.1 (3.6)	53.0-67.2	57.9 (3.5)	51.0-64.7	.19	0.62
	Satisfaction of social role	40.1 (2.5)	35.2-45.0	42.9 (2.6)	37.7-48.1	.09	1.10^b^
	Pain interference	66.7 (2.2)	62.3-71.0	63.1 (2.1)	58.9-67.2	.003^a^	1.67^b^
**Subscales (0-10)**							
	Pain intensity	7.0 (1.5)	6.60-7.49	6.5 (2.2)	5.75-7.20	.07	0.27
**Subscales (8-50)**							
	Pain impact	33.95 (7.4)	19.5-48.3	30.61 (8.5)	13.9-47.3	.007^a^	0.42

^a^Paired *t* test was used for depression, which was normally distributed. Regressions were applied to calculate *P* values for other subscales.

^b^The result is statistically significant at .05 level.

^c^The result is of a large effect size (Cohen d>0.8).

Table 4 shows the results of HEIQ among all the participants. There was a medium group-change effect size for skill and technique acquisition subscale (0.51). There was a small group-change effect size for health-directed behavior (0.35), positive and active engagement in life (0.28), and social integration and support (0.20). Net positive changes were seen for all domains. There was an increase in pain self-efficacy from baseline to 9 weeks (risk ratio [RR] 1.21 [95% CI 1.10-1.34]; *P*=.0001). No difference was seen in perceived stress score (RR 0.95 [95% CI 0.83-1.09]; *P*=.47).

**Table 4:** Health Education Impact Questionnaire results for all participants in Our Whole Lives for Chronic Pain study (N=35).

Subscale names	Baseline mean	Follow-up mean	Mean change	Group-change effect size^a^	Percent with reliable increase (%)	Percent with reliable decrease (%)	Net positive change (%)
Health-directed behavior	2.72	2.94	0.22	0.35	19	3	17
Positive and active engagement in life	2.99	3.17	0.15	0.28	19	6	14
Self-monitoring and insight	3.12	3.21	0.07	0.17	19	11	8
Constructive attitudes and approaches	3.09	3.16	0.04	0.08	11	8	3
Skill and technique acquisition	2.82	3.07	0.25	0.51	28	6	22
Social integration and support	2.84	2.98	0.12	0.20	19	6	14
Health services navigation	3.17	3.26	0.09	0.19	14	8	6
Emotional distress^b^	2.54	2.40	−0.10	−0.16	11	8	3

^a^Percentages are the proportions of participants who exceeded the threshold for reliable change.

^b^Percentages for emotional distress are reversed—the proportions in the positive reliable change cell are of those participants who had a reliably greater negative score on emotional distress at follow-up.

The medication use values of participants are presented in Table 5. At baseline, 74% (32/43) of all participants had any pain medication use in last 7 days, which increased to 83% (30/36) at 9 weeks (OR [95% CI]=1.69 [0.72-3.97], *P*=.23). Of these, 44% (19/43) used opioids at baseline, which decreased to 31% (11/36) at 9 weeks (OR [95% CI]=0.61 [0.39-0.94], *P*=.03). This was a statistically significant reduction in opioid use. In all, 51% (22/43) had used NSAIDs at baseline, which decreased to 44% (16/36) at 9 weeks (OR [95% CI]=0.73 [0.42-1.29], *P*=.28). There was no significant difference for other pain medication use between baseline (20/43, 47%) and 9 weeks (19/36, 53%) (OR [95% CI]=1.21 [0.60-2.46], *P*=.59).

**Table 5:** Pain medication use in the last week.

Pain medication	Baseline total (N=43)^a^, n (%)	9-week total (N=36), n (%)
Medication use	32 (74)	30 (83)
Opioid use	19 (44)	11 (31)
Nonsteroidal anti-inflammatory drug use	22 (51)	16 (44)
Miscellaneous/other medication use	20 (47)	19 (53)

^a^A total of 11 participants did not use medication at baseline.

### Our Whole Lives Use and Attitude Toward Computer Results

For results of participants’ attitudes toward computers, two subscales—comfort and efficacy—were used. For the entire sample, there were no statistically significant increases for either comfort or efficacy (*P*=.53 and .57, respectively).

The time (in minutes) that participants spent on each activity from the OWLCP website across 9 weeks was noted. For a participant, the average number of minutes of use of OWLCP was 659 min (minimum=2, maximum=2352). The average numbers of days of use was 19 days (minimum=1 day, maximum=63 days). On average, participants spent a total of 61 min completing body scan, 45 min watching health topic videos, 25 min performing other meditation (AOB meditation, sitting meditation, or loving kindness meditation), and 24 min watching yoga videos. The mean number of log-ins per person was 25 (SD 24.9).

For the community blog, there were 348 posts involving 27 participants—average of 14 posts per person (minimum 0, maximum 51; 64% of the total sample), and for the private journal, there were 122 posts involving 27 participants (64% of the total sample; see Multimedia Appendix 1 for community board themes). No adverse events were reported.

## Discussion

This is the first study to test an online clinician-monitored stand-alone self-management MBI system among urban diverse patients with chronic pain. We found a statistically significant increase in pain self-efficacy and a reduction in pain interference and depression. The average pain impact score decreased significantly, and the opioid use saw a 13% reduction. There were no significant changes from baseline to follow-up for physical function, anxiety, fatigue, and sleep disturbance. In terms of health education impact, there were increases in skill and technique acquisition, health-directed behavior, positive and active engagement in life, and social integration and support. Thus, we showed it is feasible for the OWLCP platform to function without an in-person medical group visit.

The findings of decreased pain interference, pain self-efficacy, and a reduction in average pain are consistent with those of studies of rural patients and international studies [50-54]. For example, Rini et al used an 8-week, automated, internet-based system called PainCoach, which included mind-body exercises in a racially diverse sample of participants with osteoarthritis in North Carolina. There was a significant pain reduction in women, and there were improvements in self-efficacy in both men and women [55,56]. In an Australian RCT of 148 participants with knee osteoarthritis, the PainCoach intervention significantly improved pain and physical function compared with the control group at 3 months. These improvements were sustained at 9 months [57]. Davis et al [51] studied an online 6-week MBI in patients with fibromyalgia, which showed an increase in self-efficacy for coping with pain.

Given the significant issues associated with chronic pain and its effect on work disability, social isolation, poor quality of life, and function, there is a great need for easily accessible culturally competent MBIs. An internet-based intervention that promotes nonpharmacological self-management and social support is an ideal approach in this population. Ziebland et al [22] noted that patients with chronic pain who made contact with others and learned from their experiences in managing pain emphasized the benefits of peer support. Furthermore, internet-delivered interventions would enhance accessibility for patients with chronic pain in rural areas that have difficulty with transportation or other physical limitations [58-61].

There have been several systematic reviews that have looked at Web-based technology for the treatment of chronic pain using nonpharmacological techniques such as MBIs [28,29,62-64]. In a systematic review of 16 studies by Toivonen et al [65], Web-based MBIs for people with chronic pain or fibromyalgia, irritable bowel syndrome, and other physical conditions showed positive effects compared with usual care on a variety of outcomes including pain acceptance, coping measures, and depressive symptoms as reported by most studies. In addition, systematic reviews have indicated that MBIs increase pain acceptance, pain tolerance, and ratings of life quality and reduce pain-associated psychological distress [66,67]. However, a more recent meta-analysis by Bawa et al [68] reviewed 11 studies that used only a randomized control group design provided less substantial effect sizes (eg, compared with control conditions) for clinical outcomes and considerable heterogeneity with regard to effect sizes. Furthermore, systematic reviews have assessed Web-supported MBIs on mental health reporting small to moderate beneficial effects of the interventions on depression, anxiety, stress, well-being, and mindfulness [69,70].

In total, 43% of our participants used opioids at baseline, which showed a statistically significant decrease from 44% to 31% at 9 weeks. However, there was an increase in any pain medication use, which increased from 74% to 83% at 9 weeks (OR [95% CI]=1.69 [0.72-3.97], *P*=.23). This was not statistically significant. This increase may be due to the increase in other types of pain medications such as acetaminophen, cyclobenzaprine, and gabapentin. For this analysis, we used self-reported data, which may have been underestimated by participants. These findings need to be reproduced in a larger fully powered RCT.

A variety of factors may contribute to the benefits gained from using OWLCP as an MBI for patients with chronic pain. First, OWLCP was designed to simulate social connections by using an interactive blog. Using an iterative development process with PAGs and extensive beta testing, OWLCP was designed for low health literacy and diverse patient populations.

Many patients use the internet/mobile phone for social support, maintaining relationships with others, and finding health information [71]. In addition, the latest technology trends in the United States indicate that mobile phone adoption rates by those experiencing the highest rates of health disparities are increasing [31-34]. A recent systematic review documented significant improvements in outcome measures related to health behavior change using internet websites [72,73].

### Limitations

Limitations to this feasibility study include a small sample size, short duration of the intervention, and self-reported information. By including only participants who had access to the internet, we may have biased the sample toward participants with favorable use of technology. This limitation could be addressed in future studies by providing a mobile phone and access to the internet. The OWLCP system was developed in English, thus excluding participants who were not fluent in English. Another limitation of the study is that it lacked a control group and had a larger sample of women compared with men. If this was a larger study, some of these limitations would be addressed. Although OWLCP recorded the number of minutes the participant was logged into the system, we do not know if the participant was actually practicing the mind-body techniques. We had no external sensors or video cameras to document objective measurement. On the flip side, we do not know if the participant was practicing outside of being logged into OWLCP.

### Conclusions

In conclusion, we found strong evidence on the feasibility of OWLCP use by low-income, racially diverse patients with chronic pain as a stand-alone intervention. OWLCP increased pain-self efficacy and reduced pain interference and pain impact. We hypothesized that the benefits include increase in skill and technique acquisition, health-directed behavior, and social integration and support. However, future studies should focus on making OWLCP more accessible by removing the barrier of internet reliability (stand-alone application) and including other languages. Future studies should also add objective measurements for OWLCP.

## Appendix

Multimedia Appendix 1 A description of the qualitative data collected from the Community Board during the intervention.

## Abbreviations

AOB: awareness of breath
ATCQ: Attitudes Toward Computers Questionnaire
BMC: Boston Medical Center
HEIQ: Health Education Impact Questionnaire
IMGV: Integrative Medical Group Visit
MBI: mindfulness-based interventions
NSAIDs: nonsteroidal anti-inflammatory drugs
OR: odds ratio
OWL: Our Whole Lives
OWLCP: OWL for Chronic Pain
PAG: patient advisory group
PCP: primary care provider
PI: primary investigator
PROMIS-29: Patient-Reported Outcomes Measurement Information System
PSS: Perceived Stress Scale
RA: research assistant
RCT: randomized controlled trial
RR: risk ratio
YMCA: Young Men’s Christian Association

## References

[1] Nahin RL (2015). Estimates of pain prevalence and severity in adults: United States, 2012. J Pain.

[2] (2019). US Department of Health and Human Services.

[3] Chou R, Turner JA, Devine EB, Hansen RN, Sullivan SD, Blazina I, Dana T, Bougatsos C, Deyo RA (2015). The effectiveness and risks of long-term opioid therapy for chronic pain: a systematic review for a National Institutes of Health Pathways to Prevention Workshop. Ann Intern Med.

[4] Alford DP (2013). Chronic back pain with possible prescription opioid misuse. J Am Med Assoc.

[5] Ray WA, Chung CP, Murray KT, Hall K, Stein CM (2016). Prescription of long-acting opioids and mortality in patients with chronic noncancer pain. J Am Med Assoc.

[6] Bohnert AS, Valenstein M, Bair MJ, Ganoczy D, McCarthy JF, Ilgen MA, Blow FC (2011). Association between opioid prescribing patterns and opioid overdose-related deaths. J Am Med Assoc.

[7] Martin BI, Deyo RA, Mirza SK, Turner JA, Comstock BA, Hollingworth W, Sullivan SD (2008). Expenditures and health status among adults with back and neck problems. J Am Med Assoc.

[8] Scherrer JF, Salas J, Copeland LA, Stock EM, Ahmedani BK, Sullivan MD, Burroughs T, Schneider FD, Bucholz KK, Lustman PJ (2016). Prescription opioid duration, dose, and increased risk of depression in 3 large patient populations. Ann Fam Med.

[9] Scherrer JF, Svrakic DM, Freedland KE, Chrusciel T, Balasubramanian S, Bucholz KK, Lawler EV, Lustman PJ (2014). Prescription opioid analgesics increase the risk of depression. J Gen Intern Med.

[10] Simon LS (2012). Relieving pain in America: a blueprint for transforming prevention, care, education, and research. J Pain Palliat Care Pharmacother.

[11] Newman AK, Van Dyke BP, Torres CA, Baxter JW, Eyer JC, Kapoor S, Thorn BE (2017). The relationship of sociodemographic and psychological variables with chronic pain variables in a low-income population. Pain.

[12] Orhan C, van Looveren E, Cagnie B, Mukhtar NB, Lenoir D, Meeus M (2018). Are pain beliefs, cognitions, and behaviors influenced by race, ethnicity, and culture in patients with chronic musculoskeletal pain: a systematic review. Pain Physician.

[13] Hirsh AT, Hollingshead NA, Ashburn-Nardo L, Kroenke K (2015). The interaction of patient race, provider bias, and clinical ambiguity on pain management decisions. J Pain.

[14] Harle C, Apathy N, Cook R, Danielson EC, DiIulio J, Downs SM, Hurley RW, Mamlin BW, Militello LG, Anders S (2018). Information needs and requirements for decision support in primary care: an analysis of chronic pain care. AMIA Annu Symp Proc.

[15] Janevic MR, McLaughlin SJ, Heapy AA, Thacker C, Piette JD (2017). Racial and socioeconomic disparities in disabling chronic pain: findings from the health and retirement study. J Pain.

[16] Escoto KH, Milbury K, Nguyen N, Cho D, Roberson C, Wetter D, McNeill LH (2018). Use of complementary health practices in a church-based African American cohort. J Altern Complement Med.

[17] Johnson C, Sheffield K, Brown R (2018). Mind-body therapies for African-American women at risk for cardiometabolic disease: a systematic review. Evid Based Complement Alternat Med.

[18] Cheng T, D'Amico S, Luo M, Lestoquoy AS, Yinusa-Nyahkoon L, Laird LD, Gardiner PM (2019). Health disparities in access to nonpharmacologic therapies in an urban community. J Altern Complement Med.

[19] Szanton SL, Wenzel J, Connolly AB, Piferi RL (2011). Examining mindfulness-based stress reduction: perceptions from minority older adults residing in a low-income housing facility. BMC Complement Altern Med.

[20] Beatty L, Lambert S (2013). A systematic review of internet-based self-help therapeutic interventions to improve distress and disease-control among adults with chronic health conditions. Clin Psychol Rev.

[21] Rini C, Williams DA, Broderick JE, Keefe FJ (2012). Meeting them where they are: using the internet to deliver behavioral medicine interventions for pain. Transl Behav Med.

[22] Ziebland S, Lavie-Ajayi M, Lucius-Hoene G (2015). The role of the internet for people with chronic pain: examples from the DIPEx International Project. Br J Pain.

[23] Andersson G, Titov N (2014). Advantages and limitations of internet-based interventions for common mental disorders. World Psychiatry.

[24] Cuijpers P, Marks IM, van Straten A, Cavanagh K, Gega L, Andersson G (2009). Computer-aided psychotherapy for anxiety disorders: a meta-analytic review. Cogn Behav Ther.

[25] Plaza I, Demarzo MM, Herrera-Mercadal P, García-Campayo J (2013). Mindfulness-based mobile applications: literature review and analysis of current features. JMIR Mhealth Uhealth.

[26] Rogers MA, Lemmen K, Kramer R, Mann J, Chopra V (2017). Internet-delivered health interventions that work: systematic review of meta-analyses and evaluation of website availability. J Med Internet Res.

[27] Wahbeh H, Svalina MN, Oken BS (2014). Group, one-on-one, or internet? Preferences for mindfulness meditation delivery format and their predictors. Open Med J.

[28] Macea DD, Gajos K, Calil YA, Fregni F (2010). The efficacy of web-based cognitive behavioral interventions for chronic pain: a systematic review and meta-analysis. J Pain.

[29] Ashraf EM (2015). Internet-based interventions for pain management: a systematic review of randomised controlled trial (RCTs) conducted from 2010 to 2014. J Public Health Epidemiol.

[30] (2019). Pew Research Center.

[31] Anderson-Lewis C, Darville G, Mercado RE, Howell S, di Maggio S (2018). mHealth technology use and implications in historically underserved and minority populations in the United States: systematic literature review. JMIR Mhealth Uhealth.

[32] Billings DW, Leaf SL, Spencer J, Crenshaw T, Brockington S, Dalal RS (2015). A randomized trial to evaluate the efficacy of a web-based HIV behavioral intervention for high-risk African American women. AIDS Behav.

[33] Joseph RP, Dutton GR, Cherrington A, Fontaine K, Baskin M, Casazza K, Lorch D, Allison JJ, Durant NH (2015). Feasibility, acceptability, and characteristics associated with adherence and completion of a culturally relevant internet-enhanced physical activity pilot intervention for overweight and obese young adult African American women enrolled in college. BMC Res Notes.

[34] Staffileno BA, Tangney CC, Fogg L (2018). Favorable outcomes using an eHealth approach to promote physical activity and nutrition among young African American women. J Cardiovasc Nurs.

[35] Berman RL, Iris MA, Bode R, Drengenberg C (2009). The effectiveness of an online mind-body intervention for older adults with chronic pain. J Pain.

[36] Gardiner P, Lestoquoy AS, Gergen-Barnett K, Penti B, White LF, Saper R, Fredman L, Stillman S, Negash NL, Adelstein P, Brackup I, Farrell-Riley C, Kabbara K, Laird L, Mitchell S, Bickmore T, Shamekhi A, Liebschutz JM (2017). Design of the integrative medical group visits randomized control trial for underserved patients with chronic pain and depression. Contemp Clin Trials.

[37] Shamekhi A, Bickmore T, Lestoquoy A, Negash L, Gardiner P (2016). Blissful agents: adjuncts to group medical visits for chronic pain and depression. Intelligent Virtual Agents: 16th International Conference.

[38] Cornelio-Flores O, Lestoquoy AS, Abdallah S, DeLoureiro A, Lorente K, Pardo B, Olunwa J, Gardiner P (2018). The Latino integrative medical group visit as a model for pain reduction in underserved Spanish speakers. J Altern Complement Med.

[39] Lee C, Crawford C, Swann S, Active Self-Care Therapies for Pain (PACT) Working Group (2014). Multimodal, integrative therapies for the self-management of chronic pain symptoms. Pain Med.

[40] Delgado R, York A, Lee C, Crawford C, Buckenmaier C, Schoomaker E, Crawford P, Active Self-Care Therapies for Pain (PACT) Working Group (2014). Assessing the quality, efficacy, and effectiveness of the current evidence base of active self-care complementary and integrative medicine therapies for the management of chronic pain: a rapid evidence assessment of the literature. Pain Med.

[41] Goode AP, Coeytaux RR, McDuffie J, Duan-Porter W, Sharma P, Mennella H, Nagi A, Williams JW (2016). An evidence map of yoga for low back pain. Complement Ther Med.

[42] Dresner D, Resnick K, Gardiner P, Barnett KG, Laird L (2014). Qualitative evaluation of an integrative medicine group visits program for patients with chronic pain and associated comorbidities. J Altern Complement Med.

[43] Gardiner P, Crooks D, Shamekhi A, McCue K, Bickmore TW (2015). Patient Advisory Groups and Integrative Medicine: How and Why to Incorporate them into Research Studies. Proceedings of the IM4US 5th Annual Conference for Integrative Medicine.

[44] Osborne RH, Elsworth GR, Whitfield K (2007). The Health Education Impact Questionnaire (heiQ): an outcomes and evaluation measure for patient education and self-management interventions for people with chronic conditions. Patient Educ Couns.

[45] Deyo RA, Ramsey K, Buckley DI, Michaels L, Kobus A, Eckstrom E, Forro V, Morris C (2016). Performance of a Patient Reported Outcomes Measurement Information System (PROMIS) short form in older adults with chronic musculoskeletal pain. Pain Med.

[46] Reznikoff M, Holland CH, Stroebel CF (1967). Attitudes toward computers among employees of a psychiatric hospital. Ment Hyg.

[47] Chen WH, Revicki DA, Amtmann D, Jensen MP, Keefe FJ, Cella D (2012). Development and analysis of PROMIS pain intensity scale. Qual Life Res.

[48] Nicholas MK (2007). The pain self-efficacy questionnaire: taking pain into account. Eur J Pain.

[49] Amtmann D, Cook KF, Jensen MP, Chen W, Choi S, Revicki D, Cella D, Rothrock N, Keefe F, Callahan L, Lai J (2010). Development of a PROMIS item bank to measure pain interference. Pain.

[50] Gardner-Nix J, Backman S, Barbati J, Grummitt J (2008). Evaluating distance education of a mindfulness-based meditation programme for chronic pain management. J Telemed Telecare.

[51] Davis MC, Zautra AJ (2013). An online mindfulness intervention targeting socioemotional regulation in fibromyalgia: results of a randomized controlled trial. Ann Behav Med.

[52] Ly KH, Trüschel A, Jarl L, Magnusson S, Windahl T, Johansson R, Carlbring P, Andersson G (2014). Behavioural activation versus mindfulness-based guided self-help treatment administered through a smartphone application: a randomised controlled trial. BMJ Open.

[53] Zernicke KA, Campbell TS, Speca M, McCabe-Ruff K, Flowers S, Carlson LE (2014). A randomized wait-list controlled trial of feasibility and efficacy of an online mindfulness-based cancer recovery program: the eTherapy for cancer applying mindfulness trial. Psychosom Med.

[54] Dowd H, Hogan MJ, McGuire BE, Davis MC, Sarma KM, Fish RA, Zautra AJ (2015). Comparison of an online mindfulness-based cognitive therapy intervention with online pain management psychoeducation: a randomized controlled study. Clin J Pain.

[55] Rini C, Porter LS, Somers TJ, McKee DC, Keefe FJ (2014). Retaining critical therapeutic elements of behavioral interventions translated for delivery via the internet: recommendations and an example using pain coping skills training. J Med Internet Res.

[56] Rini C, Porter LS, Somers TJ, McKee DC, DeVellis RF, Smith M, Winkel G, Ahern DK, Goldman R, Stiller JL, Mariani C, Patterson C, Jordan JM, Caldwell DS, Keefe FJ (2015). Automated internet-based pain coping skills training to manage osteoarthritis pain: a randomized controlled trial. Pain.

[57] Bennell KL, Nelligan R, Dobson F, Rini C, Keefe F, Kasza J, French S, Bryant C, Dalwood A, Abbott JH, Hinman RS (2017). Effectiveness of an internet-delivered exercise and pain-coping skills training intervention for persons with chronic knee pain: a randomized trial. Ann Intern Med.

[58] Winzelberg AJ, Classen C, Alpers GW, Roberts H, Koopman C, Adams RE, Ernst H, Dev P, Taylor CB (2003). Evaluation of an internet support group for women with primary breast cancer. Cancer.

[59] Yanez B, McGinty HL, Mohr DC, Begale MJ, Dahn JR, Flury SC, Perry KT, Penedo FJ (2015). Feasibility, acceptability, and preliminary efficacy of a technology-assisted psychosocial intervention for racially diverse men with advanced prostate cancer. Cancer.

[60] Penedo FJ, Dahn JR, Molton I, Gonzalez JS, Kinsinger D, Roos BA, Carver CS, Schneiderman N, Antoni MH (2004). Cognitive-behavioral stress management improves stress-management skills and quality of life in men recovering from treatment of prostate carcinoma. Cancer.

[61] Duffecy J, Sanford S, Wagner L, Begale M, Nawacki E, Mohr DC (2013). Project onward: an innovative e-health intervention for cancer survivors. Psychooncology.

[62] Garg S, Garg D, Turin TC, Chowdhury MFU (2016). Web-based interventions for chronic back pain: a systematic review. J Med Internet Res.

[63] Bender JL, Radhakrishnan A, Diorio C, Englesakis M, Jadad AR (2011). Can pain be managed through the internet? A systematic review of randomized controlled trials. Pain.

[64] Heapy AA, Higgins DM, Cervone D, Wandner L, Fenton BT, Kerns RD (2015). A systematic review of technology-assisted self-management interventions for chronic pain: looking across treatment modalities. Clin J Pain.

[65] Toivonen KI, Zernicke K, Carlson LE (2017). Web-based mindfulness interventions for people with physical health conditions: systematic review. J Med Internet Res.

[66] Chiesa A, Serretti A (2011). Mindfulness-based interventions for chronic pain: a systematic review of the evidence. J Altern Complement Med.

[67] Veehof MM, Oskam M, Schreurs KM, Bohlmeijer ET (2011). Acceptance-based interventions for the treatment of chronic pain: a systematic review and meta-analysis. Pain.

[68] Bawa FL, Mercer SW, Atherton RJ, Clague F, Keen A, Scott NW, Bond CM (2015). Does mindfulness improve outcomes in patients with chronic pain? Systematic review and meta-analysis. Br J Gen Pract.

[69] Spijkerman M, Pots W, Bohlmeijer E (2016). Effectiveness of online mindfulness-based interventions in improving mental health: a review and meta-analysis of randomised controlled trials. Clin Psychol Rev.

[70] Zhao D, Lustria ML, Hendrickse J (2017). Systematic review of the information and communication technology features of web- and mobile-based psychoeducational interventions for depression. Patient Educ Couns.

[71] Ziebland S, Wyke S (2012). Health and illness in a connected world: how might sharing experiences on the internet affect people's health?. Milbank Q.

[72] Vance K, Howe W, Dellavalle RP (2009). Social internet sites as a source of public health information. Dermatol Clin.

[73] Maher CA, Lewis LK, Ferrar K, Marshall S, de Bourdeaudhuij I, Vandelanotte C (2014). Are health behavior change interventions that use online social networks effective? A systematic review. J Med Internet Res.

