# Supplementary material for: An Innovative Electronic Health Toolkit (Our Whole Lives for Chronic Pain) to Reduce Chronic Pain in Patients With Health Disparities: Open Clinical Trial
Source: JMIR Mhealth Uhealth. 2020 Mar 30;8(3):e14768. doi: 10.2196/14768 (PMC7154936; doi:10.2196/14768)
Supplement: Multimedia Appendix 1 [file mhealth_v8i3e14768_app1.docx]

**Appendix 1 Community board themes posted on the OWL platform**

| **Increase in physical function** |
| --- |
| “Finally had a chance to get out for a walk on Sunday; it really felt good. Also had yoga practice and aquatic therapy yesterday. Today I feel pretty good, both mentally and physically.” – **Participant 10** |
| “I try to do a little stretching in the morning to get me going otherwise I am so stiff. Also, the thing that I have to do most is try to keep moving, even when I don't feel like it. I have even tried to do some chair exercises while watching T.V.” – **Participant 1** |
| “On other days I do my PT exercises or try and lift some free weights. Just keeping moving helps me to feel more energized.” – **Participant 8** |
| **Reduction in depression** |
| “When I am feeling overwhelmed and unbalanced I sometimes take a moment alone to let myself feel gratitude for everything I feel lucky for, and it helps me shift my emotions and refocus. It's often in these mindful moments that I become more aware of my physical pain, which often contributes to my mood. And stress can exacerbate the pain, so it can be a vicious cycle when left unchecked.” – **Participant 22** |
| “Letting go of resentments, saying I'm sorry when I'm wrong, working on being less judgmental and listening with interest to what others are saying as opposed to always needing to interject my opinion appears to really help me to stay positive and less focused on me.” – **Participant 25** |
| “…This can be a challenge for me, trying to keep my mind occupied first from negative things/ people during the winter especially. I really don't like having to put on a lot of clothes, coats etc. Most importantly, I have to find creative things to do during the winter, you are inside most of the time. Listening to music is very soothing to me as well as my journal writing.” – **Participant 1** |
| “Eating healthy helps me so much with both chronic pain and depression.” – **Participant 4** |
| “When I am feeling depressed, I try to help someone else in need. I find focusing on someone else and helping them takes the attention off of my issues and self-pity, and feel fulfilled in making a positive impact on others.” – **Participant 10** |
| “I just stayed inside today and listen to quiet music and tried to stay off the phone and rest my mind as this helped to ease some of the pain.” – **Participant 1** |
| “Reading, poetry, light yoga, singing (both on and off key) meditation, mindful moments and mindful meals to slow down the stress my body tries to hold on to due to chronic pain.” – **Participant 3** |
| **Non-Pharmacological use to reduce in chronic pain** |
| “I used to keep a notebook by my bed and every night I would try to write down three good things that happened that day, no matter how small or trivial. At night is when my pain is the worst and I feel most discouraged. Focusing on the daily positives before bed did actually make me feel a little better. I would like to start doing this again.” – **Participant 26** |
| “I try to sit outside when the sun is shining. The sun on my legs and back relieves some of the pain. I also try to meditate outside in warm sunny days I find I am able to move away from the pain and scattered thoughts.” – **Participant 15** |
| “To make a story short I went to get a massage yesterday and it's was grate my stress level went way down I watch the self-massage video and try the things it said and it help so much I found my stress buster.” – **Participant 18** |
| “Hi, I got a lot of good information on managing my stress and anxiety, the yoga and meditation practices helped a lot to relax but pain levels have not changed. I got a lot out of the mindful eating as well but also did not lose weight I feel the study helped me identify ways to deal with the pain anxiety and depression that I have to live with. I only wish I could continue being able to access the practice sessions for chair yoga and meditations as well as body scans” – **Participant 15** |
| “Yes I did get a lot out of the OWL. I am going to continue using the website here to post a new activity I just began with the info I got from the OWL study.” – **Participant 4** |
